# Schwannoma of the descending loop of the hypoglossal nerve: case report

**DOI:** 10.11604/pamj.2020.36.73.23500

**Published:** 2020-06-08

**Authors:** Nadia M´jahad, Mohamed Ridal, Houda Chafai, Amal Douida, Hammas Naoual, Aamal Akamar, Mustapha Maaroufi, Mohamed Nourredine EL Alami

**Affiliations:** 1 Faculty of Medicine and Pharmacy, Sidi Mohammed Benabdellah University, Fez, Morocco,; 2 Department of Otorhinolaryngology (ENT) and Head - Neck Surgery, Hassan II University Hospital, Fez, Morocco,; 3 Faculty of Medicine and Pharmacy, “Anatomy, Surgery and Anesthesiology” laboratory Sidi Mohammed Benabdellah University, Fez, Morocco,; 4 Department of Pathology, Hassan II University Hospital, Fez, Morocco,; 5 Department of Radiology, Hassan II University Hospital, Fez, Morocco

**Keywords:** Schwannoma, hypoglossal nerve, descending loop

## Abstract

Schwannomas of the descending loop of the hypoglossal nerve are very rare. Existing literature of the schwannoma of the descending loop of the hypoglossal nerve is limited to two previously reported case. They are slow-growing tumors that may masquerade a carotid body tumor. We herein described a rare case of schwannoma of the descending loop of the hypoglossal nerve in the s right latero-cervical region with diagnostic imaging and histopathological findings. A 37-years-old woman has had a palpable firm, mobile mass in the right latero-cervical region, of imaging, MR images showed homogeneous hypointensity on T1-weighted imaging (T1-WI), heterogeneous hyperintensity on T2-WI, and heterogeneous enhancement on contrast-enhanced T1-WI. Diagnostic imaging using computed tomography (CT) and magnetic resonance imaging (MRI) was suspected of Chemodectoma or neurogenic tumor. At operation, a 4 cm mass arising from the descending loop of the hypoglossal nerve of was resected en bloc with the loop itself; Final diagnosis was confirmed on the basis of histopathological finding and intraoperative findings. Postoperative course was uneventful and the patient is free from disease recurrence at tree-year follow-up. En bloc resection remains the real curative treatment of Schwannomas, ensuring unlimited freedom from disease, although causing functional impairment which may be significant. Nonetheless recurrence should be prevented as, besides requiring reintervention, it may harbor a malignant evolution towards sarcoma. Schwannomas of the descending loop of the hypoglossal nerve may masquerade a chemodectoma of the carotid bifurcation and can be curatively resected without any functional impairment. This case confirmed the differential diagnosis on the basis of the intraoperative finding that the tumor was continuous with the hypoglossal nerve.

## Introduction

Schwannomas are benign, slow-growing tumors of the neural sheath, which may affect cranial, sympathetic and peripheral nerves [1-5]. Approximately 25 to 45% of extracranial schwannomas occur in the head and neck [4,6], most likely around the glossopharyngeal space and the carotid artery sheath. Although occurrence in other sites is relatively rare, it has been reported to occur in the parotid gland, nose, paranasal sinuses, and the oral cavity [7- 9]; whereas schwannomas arising from the descending loop of the hypoglossal nerve are extremely rare [1, 10, 11]. In the present report, we described a case of schwannoma arising from the descending loop of the hypoglossal nerve in the right submandibular region with imaging and histopathological findings, masquerading a tumor of the carotid body.

## Patient and observation

**Figure 1:**
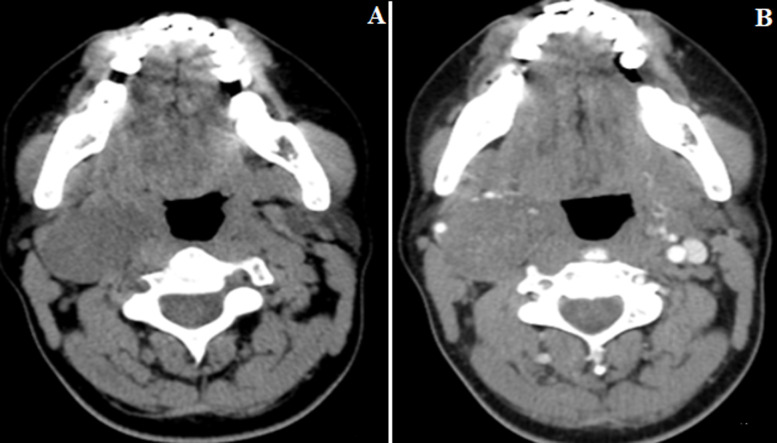
: non-contrast (A) and contrast-enhanced (B) CT images; the mass was located lateral to the right submandibular region contrast-enhanced CT demonstrated enhancement of intratumoral blood vessels

**Figure 2:**
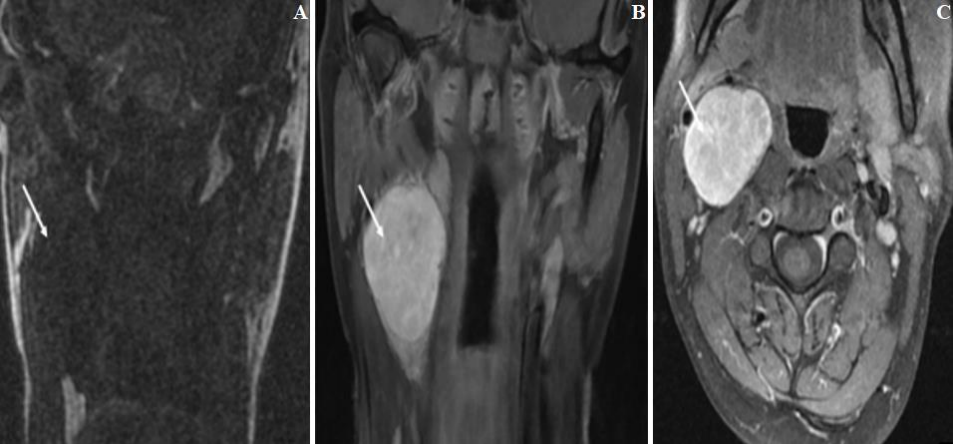
: MR images showed masse was located later to the right submandibular region (arrow), homogeneous hypo intensity on T1-WI (A), heterogeneous hyper intensity on T2-WI (B), and heterogeneous enhancement on contrast-enhanced T1-WI (C)

**Figure 3:**
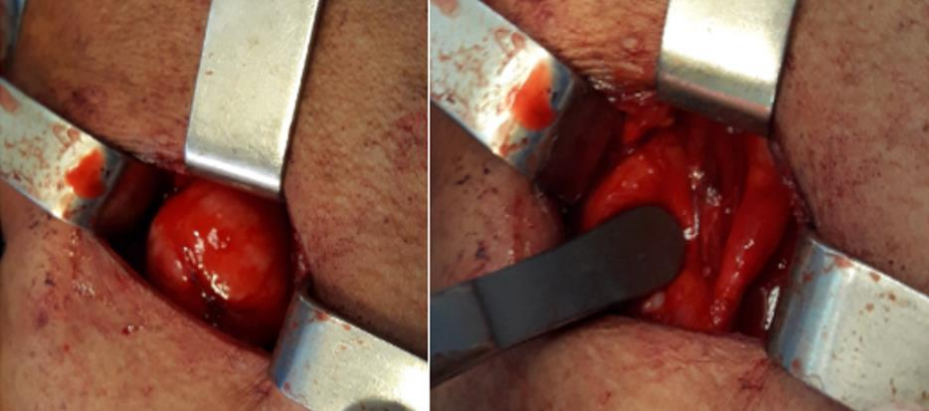
: intraoperative, the tumor was well-encapsulated and continuous with the descending branch of the hypoglossal nerve

A 37-year-old woman was referred for a non-tender, laterocervical mass enlarging within a month, without pain, dysphagia, dysarthria or any other symptom. At physical examination revealed a palpable firm, freely mobile mass measuring about 4 cm diameter, was not pulsating. Overlying skin was normal and no deviation, fasciculation or hemiatrophy of the tongue upon protrusion was observed. No lymph nodes were palpable. At cervical ultrasound examination (US), it appeared solid, heterogeneous and located between the internal (ICA) and external (ECA) carotid arteries, with normal flow velocities in both of them. Non-contrast CT revealed a well-circumscribed spherical 4,8x3,8x3,2 cm mass with heterogeneous density in the right laterocervical region. Contrast-enhanced CT demonstrated inhomogeneous weak enhancement, and intratumoral blood vessels were strong enhancement. No evidence of cystic or necrotic degeneration was present in the CT (Figure 1). One weeks later, the patient underwent MR images, MR images showed homogeneous hypointensity on T1-weighted imaging (T1-WI), heterogeneous hyperintensity on T2-WI, and heterogeneous enhancement on contrast-enhanced T1-WI. With normal flow velocities in the internal and external carotid arteries (Figure 2), the lesion was suspected of neurogenic tumor on the basis of the above imaging findings. A fine needle aspiration biopsy was not performed, due to the risk of inadvertent arterial puncture and the sufficiently clear imaging indicating surgical excision in any case. Total excision of the tumor was performed under general anesthesia. At surgery, access was gained through a standard pre-sternocleidomastoid incision. The common carotid artery (CCA), ICA and ECA were controlled at distance from the mass and the hypoglossal nerve was identified and exposed along all its course crossing the carotid bifurcation. Intraoperatively, the tumor was wellen capsulated and delimited, arose from the descending branch of the hypoglossal nerve and was easily detached from the ICA and ECA. En bloc resection of the mass together with the descending branch of the hypoglossal nerve was performed (Figure 3). The post- operative course was uneventful and no neural deficit was observed. Histopathologic examination of the surgical specimen demonstrated a central mucous matrix and the surrounding nerve axon. The specimen also contained Antoni type A and Antoni type B. Antoni type A is composed of spindle shaped Schwann cells with elongated nuclei arranged in streams and nuclear palisades known Verocay bodies. Antoni type B consists of less spindle cells and a myxoid background (fig 4), it was also characterized by positive immunohistochemical staining of S-100 protein and vimentin. Thus, the definitive diagnosis was schwannoma of the hypoglossal nerve. Regular controls, both clinical and with cervical US every 6 months on an outpatient basis were programmed. She is well and free from any disease recurrence at tree year follow-up.

## Discussion

Schwannomas are benign tumors. They usually develop from the sensory component of cranial nerves, most commonly from 8^th^ and 10^th^ cranial nerves. Hypoglossal nerve schwannoma is rare, since this nerve is purely motor. Schwannomas of the vagus and hypoglossal nerve are known to potentially masquerade a tumor of the carotid body at preoperative imaging, due to the close situation of these structures [12-16]. However, true schwannomas arising from the hypoglossal nerve are usually associated with hemiatrophy, fasciculation and deviation of the tongue upon protrusion [15], which can be rarely caused also by schwannomas of the cervical vagus nerve [16], whereas carotid body tumors are almost never associated with such symptoms. Schwannomas of the descending branch of the hypoglossal nerve should be neurologically asymptomatic, until compression of adjacent cranial nerves occurs, given the absence of functional actions of the descending branch itself, which was the case in this presentation. However, as well as tumors arising from the hypoglossal and vagus nerve, they may masquerade a carotid body tumor at diagnostic imaging [1]. This too occurred in the case object of this report. Histopathological examination demonstrates two types of schwannomas cells: Antoni type A and Antoni type B. The Antoni type A are spindle-shaped cells with parallel rows of palisading nuclear organized in whorls and waves, while the Antoni type B consists of spindle cells haphazardly scattered in a delicate, fibrillar microcystic matrix. Most schwannomas contain a mixture of both Antoni type A and Antoni type B tissue [17]. As for Schwannomas arising from other cranial nerves, excision of the mass en block with the whole nerve is the best option in order to achieve a curative treatment [3], given that, limited to the descending loop of the hypoglossal nerve, this purpose can be reached without any functional impairment. The need for a curative resection is supported by the opportunity of avoiding a redo operation in case of recurrence and by the possibility that both primary and recurrent Schwannomas may potentially show an evolution towards sarcoma [3, 13, 18-20]. Technical tips of the reported case include the standard access through a presternocleidomastoid incision, as for a standard carotid endarterectomy, a separate control of CCA, ECA and ICA together with systemic heparinization, should the need for clamping of the carotid bifurcation arise. For large masses, extending high, above C1, as anticipated at preoperative imaging, nasal intubation should be considered, in order to gain sufficient exposure toward the base of the skull and for gaining safe control of the distal ICA. Due to its anatomical situation and to the small diameter of the Schwannoma reported herein, this adjuct maneuver was, obviously, not necessary. No matter from which nerve the Schwannoma arises, regular controls, both clinical and US, on a 6-month basis for at least the first 3 years, then on a yearly basis, are strongly advised, in order to promptly detect and treat any eventual recurrence, although this is expected to be extremely rare after a proper curative resection [1].

## Conclusion

Extra cranial hypoglossal schwannoma is very rare and few cases have been described in the literature. Existing literature of the schwannoma of the descending loop of the hypoglossal nerve is limited to two previously reported case, and this report adds that prompt resection of even small masses involving the carotid bifurcation is always indicated in order to obtain the maximum possibilities of a durable cure, limiting at maximum functional impairment. Despite the rarity of schwannomas, this condition should be considered in differential diagnoses for masses localised in the neck.
